# Multifunctional Flexible Hard Coatings with Weathering Resistance and Heat-Shielding Properties

**DOI:** 10.3390/polym17040519

**Published:** 2025-02-17

**Authors:** Yuxi Chen, Shenglan Tian, Jincheng Ruan, Ruyu Chen, Lijie Qu, Luming Li

**Affiliations:** 1 College of Chemistry and Materials Engineering, Zhejiang A & F University, Hangzhou 311300, China; 19550177296@163.com (Y.C.); tianshenglan2024@163.com (S.T.); 2 Department of Product Research & Development, DE & E Holding Group, Hangzhou 311231, China; 15888165968@163.com (J.R.); chenruyu271@126.com (R.C.)

**Keywords:** flexible hard coating, hydrophobicity, heat shielding, weathering resistance

## Abstract

Hard, flexible, transparent, and hydrophobic multifunctional coatings have a wide range of applications, but they do not adequately protect against harsh conditions, especially photoaging. In this study, SiO_2_ and Al_2_O_3_ nanoparticles were first modified by silazane and epoxy-functionalized silanes and then reacted with a polyetheramine curing agent to prepare highly crosslinked multifunctional hybrid coatings at room temperature. Due to the integration of siloxane nanoparticles and a polymer network, the multifunctional coatings presented outstanding hardness (4H), flexibility (bending diameter of 10 mm), and transmittance (>97%). The introduction of low-surface-energy PDMS and methyl-rich HMDS endowed the coatings with good hydrophobicity (water contact angle = 141.37°). The high reflectivity of SiO_2_ and Al_2_O_3_ in the solar spectral region can help prevent photoaging of the coatings, improve their heat-shielding effect, and broaden their application scenarios. Compared with the traditional manufacturing methods, this study did not need ultraviolet irradiation, and the multifunctional transparent coatings could be prepared through a simple and efficient step-by-step strategy.

## 1. Introduction

Flexible yet hard coatings are used in flexible displays, lighting equipment, and energy-saving windows [1], where their superior flexibility makes them elastic with a high tolerance to bending, while their excellent hardness protects the coating and substrate from scratches [2,3]. However, in most cases, these two properties are mutually exclusive. Organic–inorganic hybrid coatings can overcome this issue by using different precursors during a sol–gel process to produce siloxane hybrid materials [4,5,6]. For example, Choi et al. synthesized a cycloaliphatic epoxy-functionalized oligosiloxane using base-catalyzed hydrolysis and the condensation of (2-(3,4-epoxycyclohexyl)ethyl) trimethoxysilane. Organic (epoxy) and inorganic (siloxane) networks were obtained from ultraviolet (UV)-initiated cationic ring-opening polymerization of the cycloaliphatic epoxy-functionalized oligosiloxane nanobuilding blocks. The highly crosslinked epoxy networks exhibited simultaneous plastic-like flexibility and glass-like wear resistance [7]. Based on this, several hybrid coatings based on cage-like polysilsesquioxanes (POSSs) with similar properties were developed. In these hybrid systems, outstanding flexibility was provided by the ring-opening polymerization of glycidyloxypropyl groups, and a superhydrophobic surface was fabricated via epoxy-amine reactions [8,9,10]. The abovementioned photocatalytic cross-linking reaction required UV irradiation and also had strict requirements for the surface morphology of the substrate, which limited the thickness of the hybrid coatings. To address this drawback, efficient preparation methods based on epoxy-amine reactions have attracted the interest of researchers. Several types of epoxy-functionalized nanoclusters were synthesized via a sol–gel approach, followed by cross-linking with various amine-terminated curing agents at room temperature. Without external UV light or heat, this step-by-step strategy can be used to develop hybrid coatings with diverse properties through the rational design of precursors [11]. Chen et al. developed a highly transparent coating by using epoxyzirconium particle and amineterminated hyperbranched polysiloxane, which exhibits excellent mechanical properties and antifouling performance. The presence of hyperbranched polysiloxane and zwitterionic silanes allows the coating to have great adhesion, hardness, flexibility, oleophobicity, and antibacterial ability [12].

Energy shortages and environmental pollution both pose serious threats to the sustainable development of human society. Flexible and hard coatings with energy-saving properties can be used for windows or walls to build envelopes that reduce the heat transfer between indoor and outdoor environments. Functionalized nanoclusters utilized in hybrid coatings must satisfy specific performance criteria, including low thermal conductance, superior UV-shielding capability, and exceptional weathering resistance to ensure long-term durability [13,14]. Excessive exposure to UV radiation can damage the molecular structure of polymeric coatings, leading to a decrease in appearance and mechanical properties [15,16]. For example, the obvious aging phenomenon of polyethylene terephthalate (PET), such as cracking and yellowing. Therefore, the use of UV-protective additives or materials is inevitable to improve the aging resistance of hybrid coatings. As reported, zinc oxide (ZnO), titanium dioxide (TiO_2_), alumina (Al_2_O_3_), and silica (SiO_2_) can provide UV protection by scattering and reflection [17]. In addition, to achieve effective heat shielding during the daytime, the design idea of passive daytime radiative cooling (PDRC) coatings can be used. The ideal material needs to meet the following two conditions: a high solar reflectivity in the wavelength range of 0.2–2.5 μm to avoid solar absorption and a high emissivity in the long-wavelength infrared atmospheric transparency window (8–13 μm) for radiating heat into cold space. Each of the nanoparticles has a high extinction coefficient in different specific regions within the atmospheric transparency window, allowing for the selective emission by designing and combining hybrid nanoparticles. As reported, SiO_2_ and Al_2_O_3_ both have high reflectivities in the wavelength range of 0.2–2.5 μm, which accounts for 95% of all solar radiation energy [18,19]. SiO_2_ and Al_2_O_3_ also have complementary emissivities in the atmospheric transparency window, which for SiO_2_ coatings is between 8 and 10 μm, and is above 11.5 μm for Al_2_O_3_ coatings. Therefore, combining SiO_2_ and Al_2_O_3_ to prepare hybrid coatings can reduce the heat input and improve the heat-shielding performance of substrates.

This study investigated highly crosslinked multifunctional hybrid coatings synthesized via the integration of sol–gel chemistry and epoxy-amine curing reactions at room temperature. Three types of nanoclusters were first modified by silazane and epoxy-functionalized silanes and then crosslinked with polyetheramine curing agents. By introducing low-surface-energy polydimethylsiloxane (PDMS) into the multifunctional hybrid coating, its hydrophobicity was further enhanced. Owing to the synergistic effects of functionalized siloxane nanoparticles and polymer networks, the hydrophobic hybrid coating showed outstanding hardness, flexibility, and transmittance, as well as weathering resistance and heat-shielding properties.

## 2. Materials and Methods

### 2.1. Materials

3-Glycidyloxypropyltrimethoxysilane (KH560), tetraethyl orthosilicate (TEOS), hexamethyldisilane (HMDS), ammonia solution, deionized water, ethanol, and polyetheramine curing agent (D400) were purchased from Shanghai Meiruier Biochemical Technology Co., Ltd. (Meiruier, Shanghai, China). PDMS (SYLGARD^®^184) was purchased from Dow Corning Co., Ltd. (Dow, Midland, TX, USA). The nano-SiO_2_ and nano-Al_2_O_3_ were purchased from Zhongzhi Advanced Materials Technology Co., Ltd. (Quanzhou, China).

### 2.2. Preparation of the Functionalized Nanoparticles

KH560 (5 mL), 10 mL of ethanol, and 2 mL of deionized water were added to a beaker, and the mixture was stirred at 30 °C for 30 min to hydrolyze the KH560.

Nano-SiO_2_ was dried in a vacuum drying oven at 100 °C for 24 h and then 2 g of dried nano-SiO_2_ was ultrasonically dispersed in 50 mL ethanol at 30 °C for 15 min. Then, it was transferred to a magnetic stirrer at 70 °C with a speed of 500 rpm. Subsequently, the hydrolyzed KH560 was slowly added and stirred for 6 h to obtain a suspension. Finally, the product was filtered and washed thoroughly with ethanol. The resulting KH560-SiO_2_ was dried at 90 °C under vacuum for 24 h. The KH560 surface-treated nano-Al_2_O_3_ powders (KH560-Al_2_O_3_) were prepared by using the same method.

TEOS (7 mL) was uniformly dispersed in 50 mL ethanol by stirring at 30 °C for 15 min, the pH was adjusted to 8 with 5% ammonia solution, and then stirring was continued for 4 h. Then, 10 mL of HMDS and 10 mL of ethanol were dispersed uniformly, added to the above SiO_2_ sol dispersion, and stirred for 2 h to obtain an HMDS-modified SiO_2_ dispersion. The resulting HMDS-SiO_2_ dispersion was cooled to room temperature and aged for 7 d.

### 2.3. Fabrication of Multifunctional Coatings

**Figure 1:**
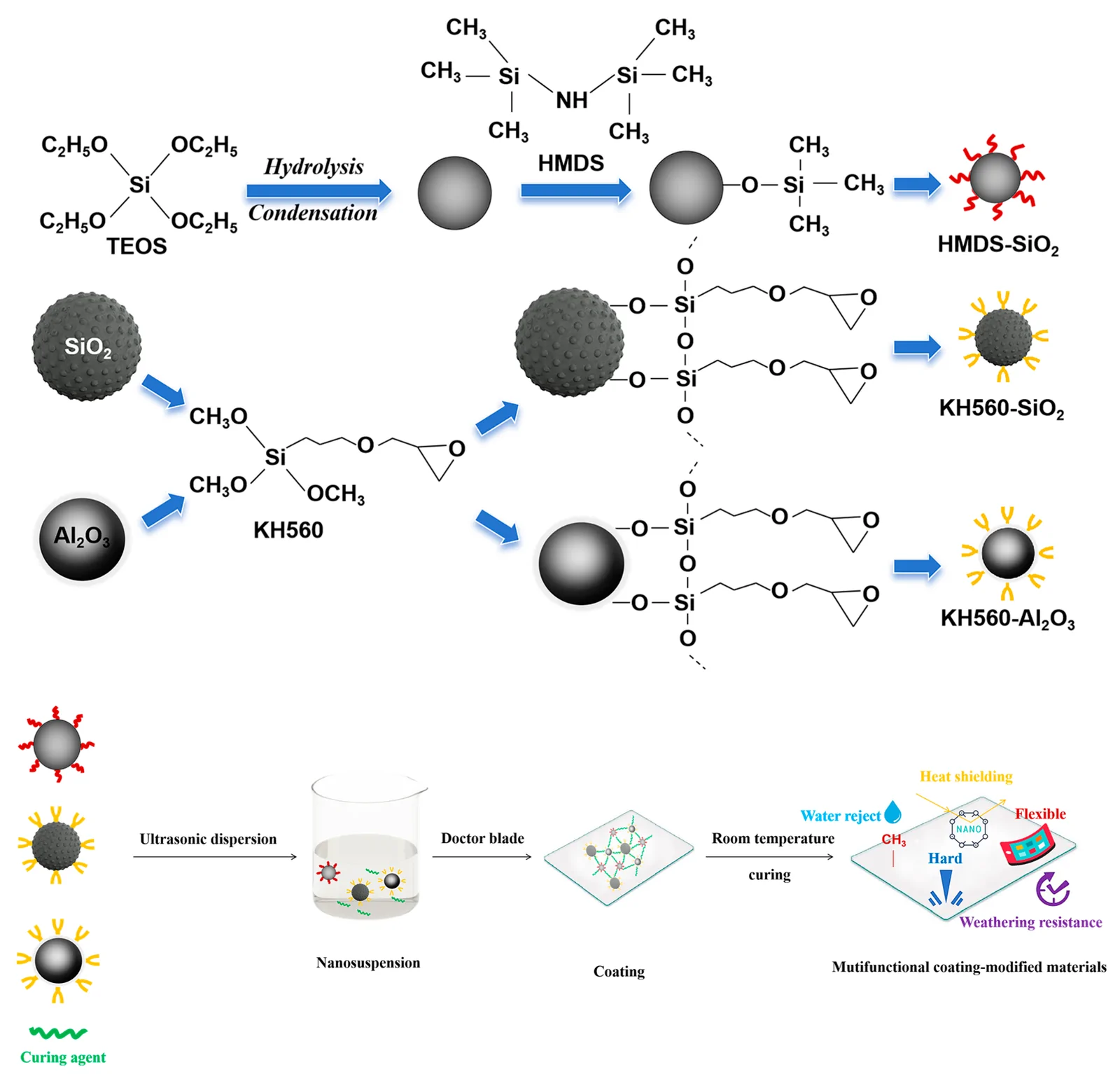
Schematic diagram for the preparation of multifunctional coatings.

The schematic diagram and formulation of multifunctional coatings are shown in Figure 1 and Table 1. First, different proportions of functionalized nanoparticles were ultrasonically dispersed in 48 mL ethyl acetate at 30 °C for 20 min to obtain a multicomponent nanoparticle suspension. Then, the uniform nanosuspension was transferred to a magnetic stirrer at 30 °C with a speed of 300 rpm. Second, 2 mL of KH-560 and 10 g of D400 were added to the nanosuspension and stirred for 30 min. The above nanosuspension was coated onto PET or glass substrates through the doctor blade method, and the coated substrates were cured at 30 °C for 7 d. PDMS was used to improve the hydrophobicity, and after 2 mL of KH560 and 7 g of D400 were added to the multicomponent nanoparticle suspension for 15 min, 3 g of PDMS and the associated curing agent were added to the nanosuspension and stirred for 15 min. The coating process was carried out using the same method mentioned above.

### 2.4. Characterization

The hardness of the multifunctional coatings was examined by a pencil hardness tester (QHQ-A, Quzhou Kehui Instrument Co., Ltd., Quzhou, China). The adhesion strengths of the coatings were tested by a cross-cutting tester (QFH-A, Quzhou Kehui Instrument Co., Ltd., Quzhou, China) based on GB/T4893.4-1985 Standard [20]. The flexibility was tested using a paint flexibility tester (QTX, Hebei Zhongke Beigong Instrument Co., Ltd., Cangzhou, China). The average surface roughness (Ra) of the coatings was measured with a surface roughness instrument (TR210, JITAI KEYI, Beijing, China).

According to GB/T1865 [21], the aging performance of the coatings was tested by a xenon lamp aging box (Atlas, Ametek Commercial Enterprise (Shanghai) Co., Ltd., Shanghai, China) for 20 d. Before and after the aging test, the coatings were assessed using an optical contact angle measuring system (OCA50AF, ShangHai YinXu Mechanical and Electrical Equipment Co., Ltd., Shanghai, China).

**Figure 2:**
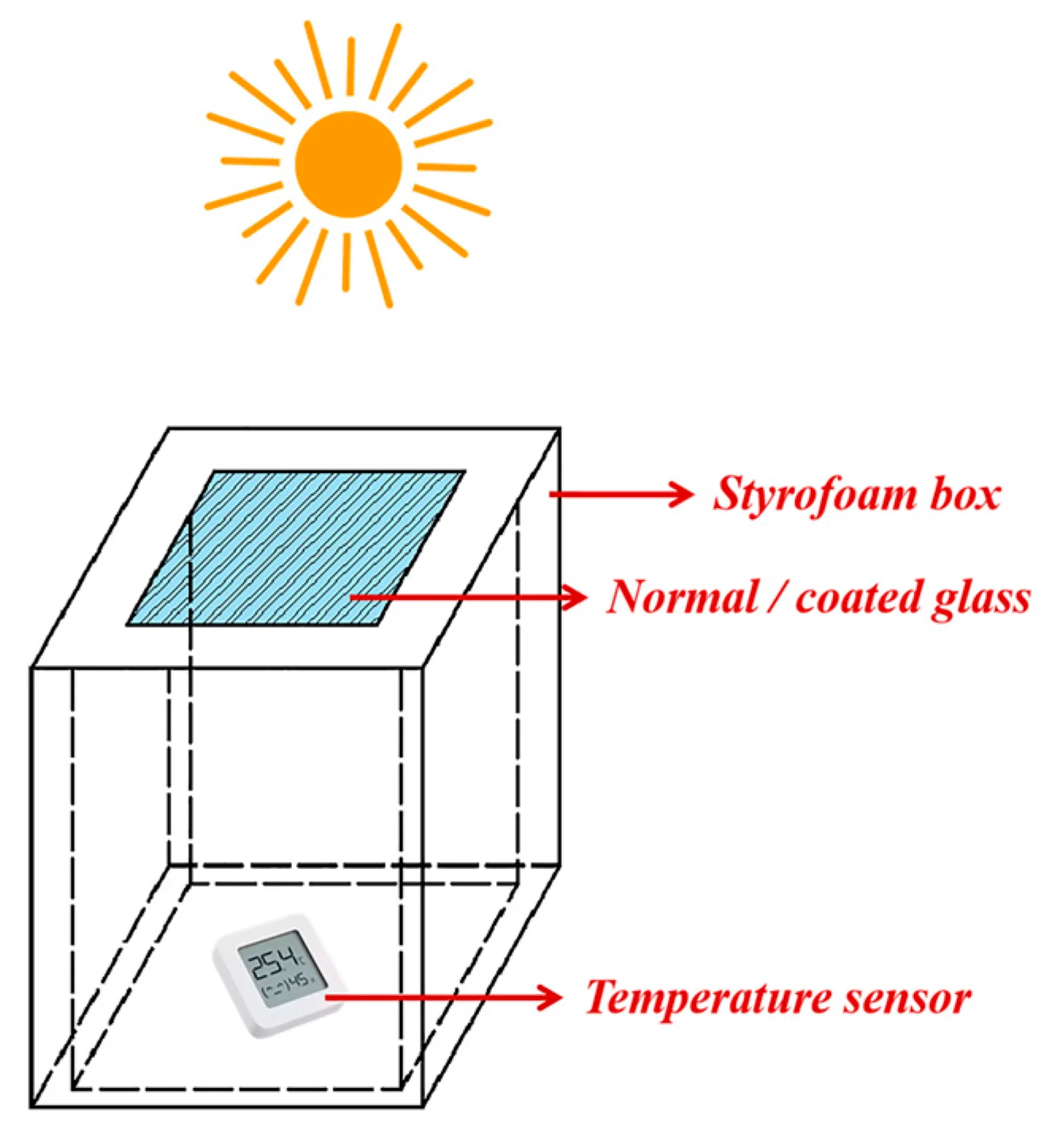
Schematic of the setup used to record the internal temperature beneath the normal and coated glasses.

The heat-shielding performance of the multifunctional coatings was evaluated by recording the temperature beneath the uncoated and coated glasses in a closed Styrofoam box (12.5 cm × 12.5 cm × 14 cm). As shown in Figure 2, the Styrofoam box had an opening of 8 cm × 8 cm at the top and was covered with uncoated glass and coated glass (thickness = 0.15 cm). A temperature sensor was fixed at the bottom surface of the box, and all custom Styrofoam boxes were placed in the same and unobstructed outdoor area.

According to ISO 22007-2 [22], the thermal conductivities of the uncoated and coated glasses in Figure 2 were measured using a thermal conductivity apparatus (Hot Disk TPS2500S, K-analysis Instrument Trading Shanghai Co., Ltd., Shanghai, China).

The reflectance spectra of the powder samples in the range of 200–2500 nm were collected by a UV-Vis-NIR spectrophotometer (UV-3600, Shimadzu (Shanghai) Experimental Equipment Co., Ltd., Shanghai, China). Functional groups of the coatings were characterized by a Fourier-transform infrared spectrometer (FT-IR, Nicolet iS20, Thermo Fisher Scientific (China) Co., Ltd., Shanghai, China) in the spectral range of 2000–500 cm^−1^, with a resolution of 4 cm^−1^.

According to ASTM D1003, the optical properties of the coated glass were characterized by measuring their transmittance and haze using a multifunctional haze meter (TH-110, Hangzhou Caipu Technology Co., Ltd., Hangzhou, China).

The chemical compositions of samples were analyzed by X-ray photoelectron spectroscopy (XPS, ESCALAB Xi+, Thermo Fisher Scientific (China) Co., Ltd., Shanghai, China).

## 3. Results and Discussion

### 3.1. Macro-Mechanical Properties of Multifunctional Coatings

**Figure 3:**
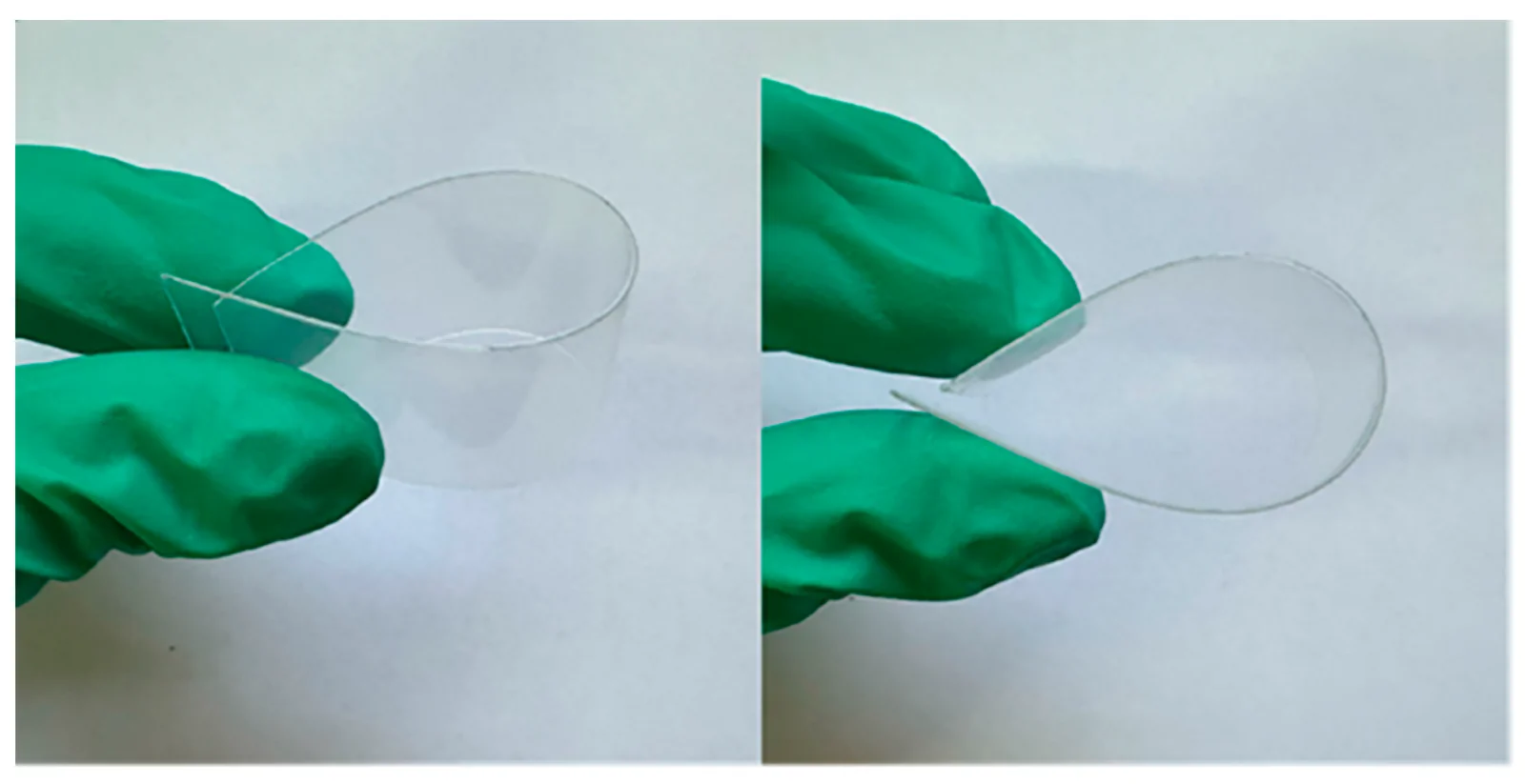
Photographs demonstrating the flexibility of the multifunctional coating on a PET substrate.

The hardness, surface roughness, and adhesion of the multifunctional coatings on the two substrates are summarized in Table 2. A higher nanoparticle content increased the hardness of the coatings in the order HAS2 = HAS3 > HAS3-PDMS > HAS1, which resulted in a higher cross-linking degree of the polymer during curing. Similar results were obtained from the adhesive strength tests. Note that the degree of adhesion grade on the PET was lower than that of glass because the adhesion is closely related to the hydrogen bonds with the substrate, and the surface of PET is non-polar. Replacing the curing agent with PDMS in the coating had a minimal impact on its mechanical properties, the hardness and adhesion of the coating decreased slightly. This was presumably due to the lower cross-linking degree of the polymer due to a reduction in the number of hydrogen bonds. The average surface roughness Ra increased with the increasing nanoparticle content, which may have been related to the aggregation of nanoclusters. The introduction of PDMS reduced the surface roughness, which could have been caused by the self-polymerization of the PDMS on the surface and its filling effect on the gaps between the nanoclusters. Moreover, the bending diameter of all the multifunctional coatings was 10 mm, indicating its good flexibility. As shown in Figure 3, a piece of 10 × 2.5 cm PET coated with multifunctional coatings was bent into a U-shape, and no cracks appeared on the coatings during the folding–unfolding process that was used 300 times.

### 3.2. Weathering Resistance of Multifunctional Coatings

**Figure 4:**
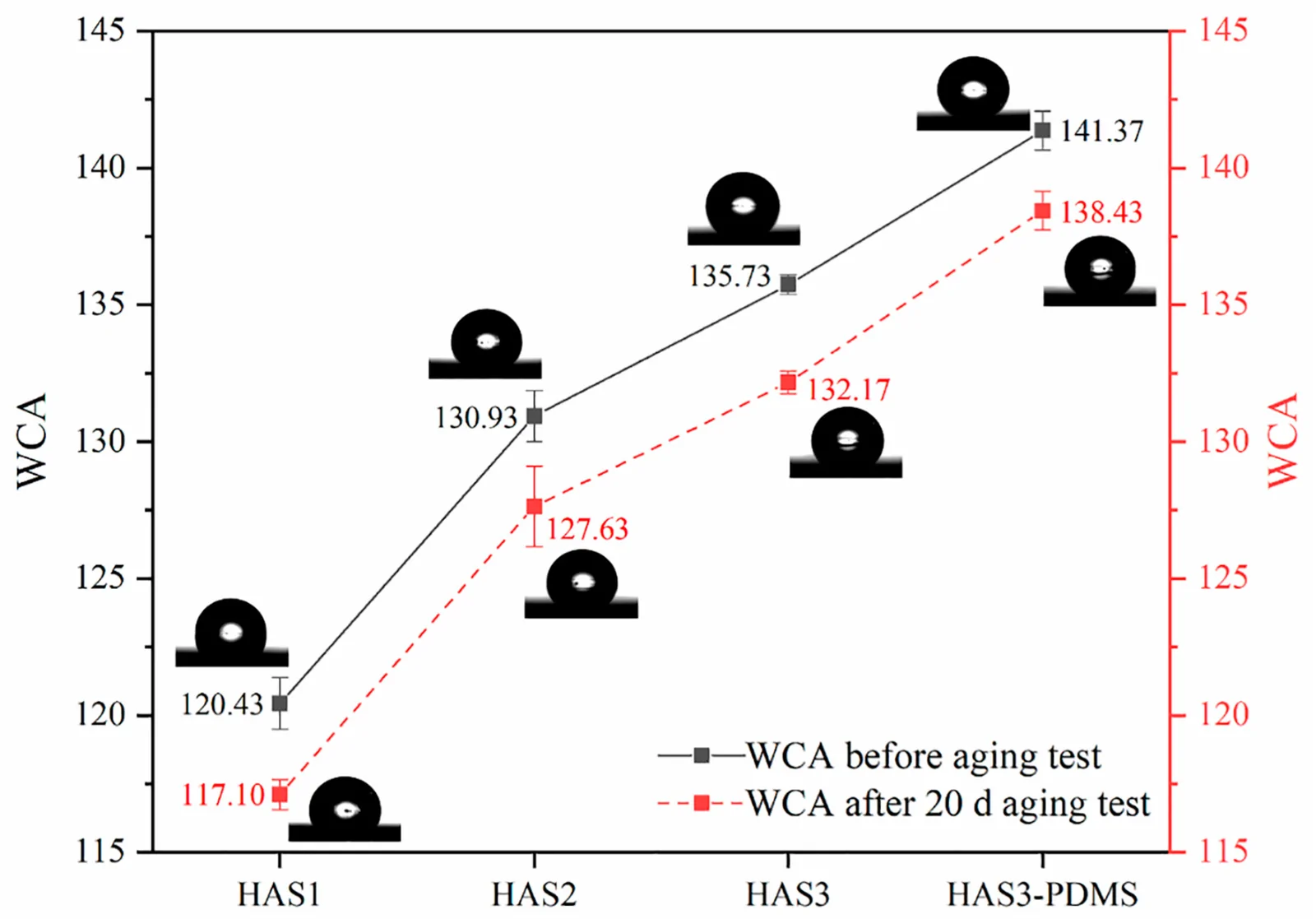
WCAs of the multifunctional coatings on a PET substrate before and after aging for 20 d.

To examine the surface wettability and weathering resistance of multifunctional coatings, the water contact angles (WCAs) of the coatings were measured before and after the aging test. The WCAs were closely related with the roughness, surface energy, and type of the functional group [23]. As shown in Figure 4, HAS1, HAS2, HAS3, and HAS3-PDMS showed WCAs of 120.43°, 130.93°, 135.73°, and 141.37° before the aging test, respectively, and the WCAs of the multifunctional coatings increased with the nanoparticle content. All the samples demonstrated excellent hydrophobic properties, with WCAs higher than 90° [24]. For HAS2, HAS3, and HAS3-PDMS (which had the same content of nanoparticles), a higher proportion of HMDS-SiO_2_ and the introduction of PDMS in the coating system resulted in a greater WCA. The greater hydrophobicity may have been due to the hydrophobic trimethylsilyl group of the HMDS and the self-enrichment of the PDMS, which has a low surface tension and facilitates liquid sliding and contraction [25,26,27].

After 20 d of aging, the WCAs decreased by 2.08–2.77%, indicating that the multifunctional coatings maintained good hydrophobicity during the test. SiO_2_ and Al_2_O_3_ endowed the multifunctional coatings with ultraviolet radiation (UV) protection by refracting and reflecting UV in the polymer matrix [28,29]. HAS3-PDMS showed the lowest drop of 2.08% because the PDMS protected the substrate by self-enrichment on the surface, and thus, improved the photoaging resistance of the multifunctional coating [30,31].

### 3.3. Heat-Shielding Property

**Figure 5:**
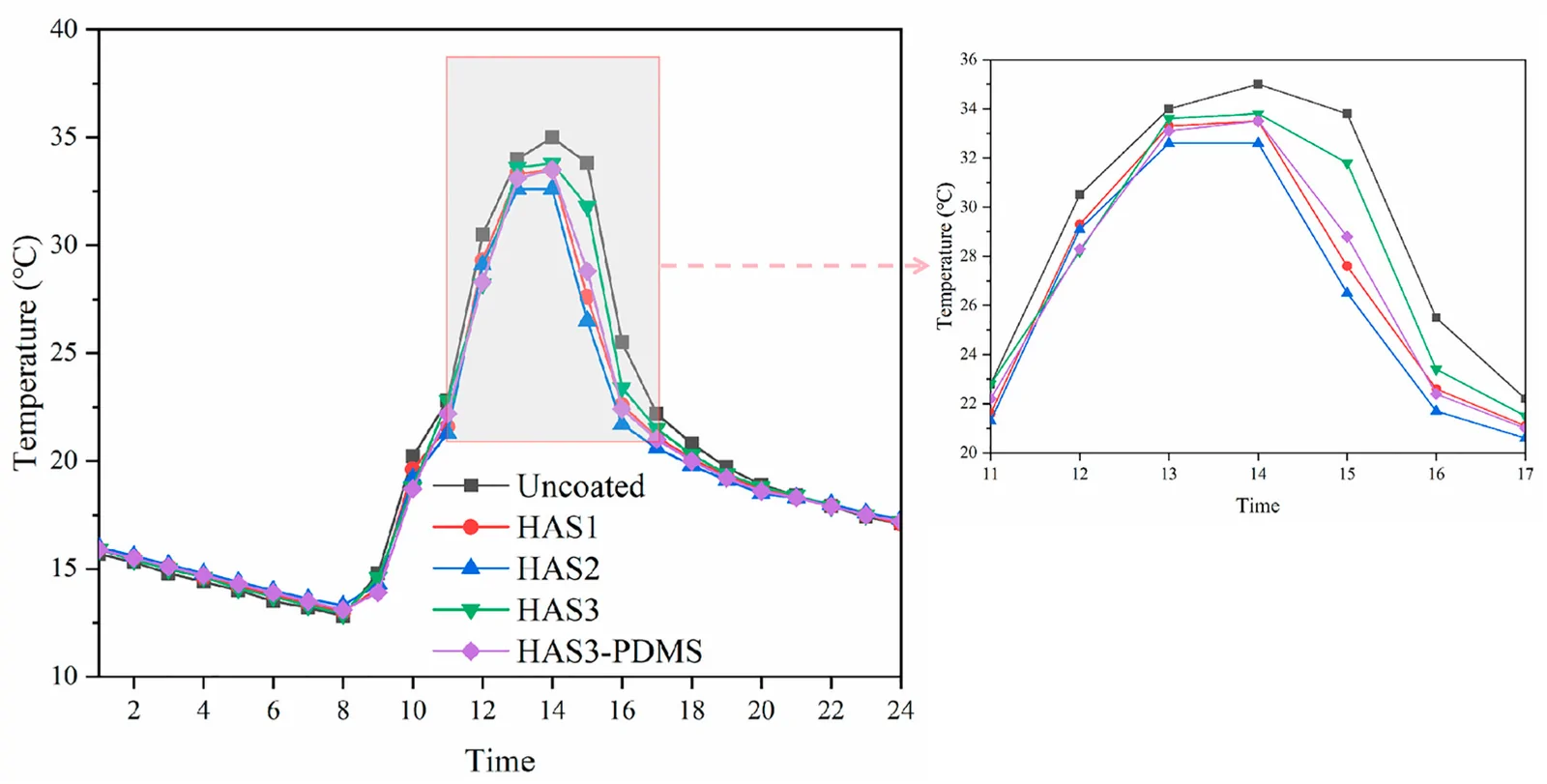
Temperature measurement result of uncoated and coated samples during the experiment (12 January).

To evaluate the heat-shielding performance of the multifunctional coatings, the curves of air temperature changes in a closed box equipped with normal and coated glasses are shown in Figure 5. The weather during the experiment was sunny, and the experimental setup was placed in an unobstructed open space facing the sky to simulate an actual application scenario. When the solar radiation was weak, the heat-shielding effect of the multifunctional coating was not significant. From 11:00 to 17:00, as the solar irradiation increased, the temperature of the coated samples rose more slowly than the uncoated sample. Even when the peak air temperature was reached at 14:00, the coated sample showed a much lower temperature, where it fluctuated between 1.2 °C and 2.4 °C. At 15:00, the temperature reductions of the HAS2, HAS3-PDMS, HAS3, and HAS1 were 7.3 °C, 6.2 °C, 5.0 °C, and 2.0 °C, respectively. These results experimentally demonstrate that increasing the content of nanoparticles and introducing the PDMS improved the heat-shielding property of the multifunctional coating. The contents of nanoparticles in the HAS2 and HAS3 samples were the same, but the temperature reduction in HAS2 was greater than that in HAS3, suggesting that increasing the proportion of Al_2_O_3_ nanoparticles had a positive effect on improving the heat-shielding property.

**Figure 6:**
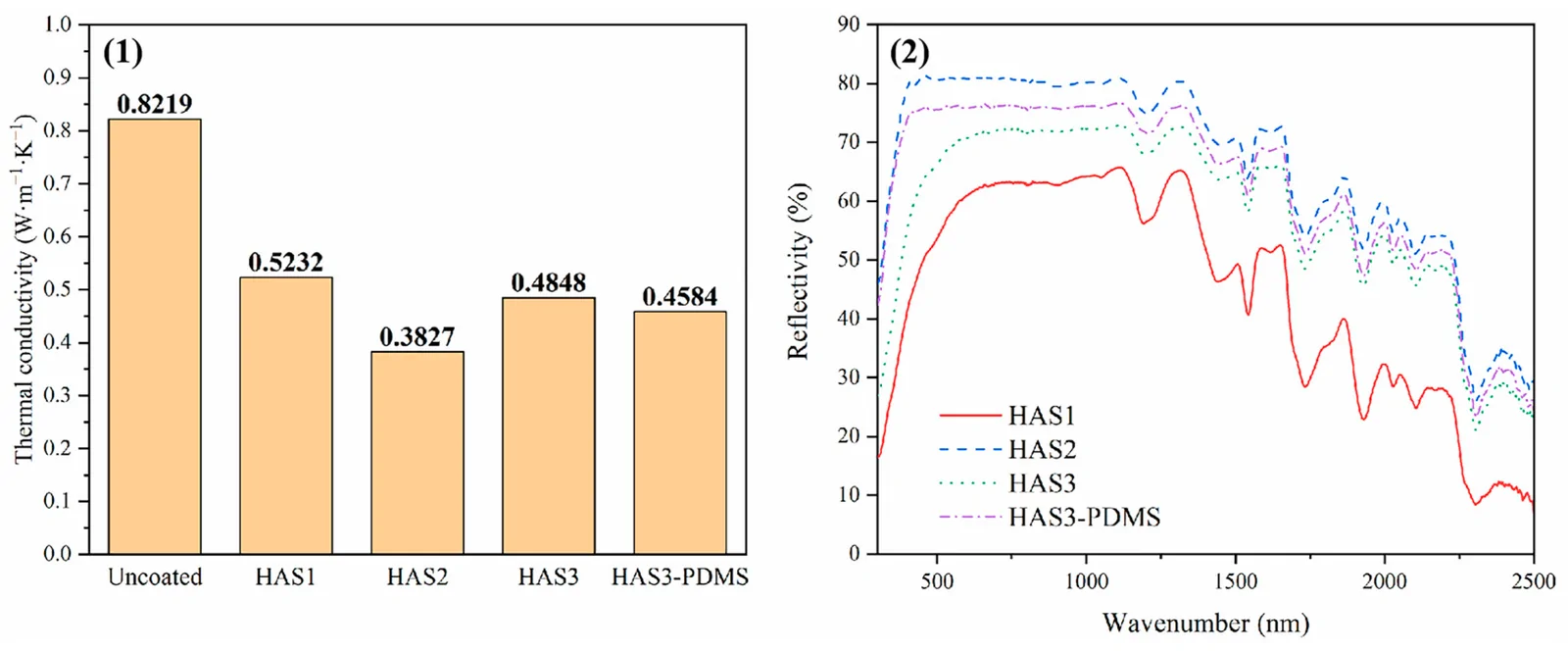
Thermal conductivity of uncoated and coated samples (**1**); FT-IR spectra of multifunctional coatings (**2**).

The thermal conductivities of commercially available uncoated glass and the multifunctional coating-modified glass are listed in Figure 6(1). Compared with the uncoated sample (0.8219 W/(m·K)), the thermal conductivities of the coated samples were about 36–53% lower, indicating that the multifunctional coatings blocked heat transfer. The reflectance values of the multifunctional coatings are shown in Figure 6(2), and the energy of the solar radiation was mainly concentrated in the ultraviolet/visible/near-infrared (UV-Vis-NIR) region of 0.3–2.5 µm [32,33]. The multifunctional coatings showed high reflectivities in this wavelength region, which could help reduce the energy absorption and improve the heat-shielding performance. The heat-shielding effect of the multifunctional coatings was likely to be affected by many factors, such as the composite ratio of the nanoparticles and the type of nanoparticles. The reflectivity of multifunctional coatings followed the order HAS2 > HAS3-PDMS > HAS3 > HAS1, which was consistent with the thermal conductivity analysis, further confirming that increasing the content of nanoparticles and introducing PDMS improved the heat shielding. This may have been attributed to the high reflectance and low thermal conductivity of SiO_2_ and Al_2_O_3_ nanoparticles. The reflection ability of Al_2_O_3_ nanoparticles may have been stronger than that of SiO_2_ nanoparticles. Moreover, PDMS has an inherently low thermal conductivity and can fill gaps between nanoparticles, thus further enhancing the heat-shielding performance of the multifunctional coatings [34].

### 3.4. Optical Properties

All of the multifunctional coatings were coated on glass with a thickness of ≈60 μm, and the transmittance of the bare glass substrate was included as a control. The transmittance and haze of the coated glass are listed in Table 3. The optical transmittance of all samples exceeded 97%, which is beneficial for ensuring a high indoor illumination and expanding the application of transparent coatings in the field of smart windows [35]. In contrast, HAS3-PDMS had the lowest transmittance, but the difference was not very large. Similar to the reported research [12], increasing the content of nanoparticles was beneficial for improving the mechanical properties of the multifunctional coatings, but the negative impact on the transmittance of the coated glass was not obvious. The haze was significantly increased with the increasing nanoparticle content and the introduction of PDMS. The haze of the coated samples was closely related to the particle size of the multifunctional coatings, which was controlled by the degree of cross-linking. The self-polymerization of the PDMS on the surface slightly reduced the surface roughness, which limited the propagation of scattered light. However, the PDMS replaced some of the curing agents, which may have caused the agglomeration of the nanoparticles. Combined with the inherent scattered light of the PDMS, the haze of HAS3-PDMS was significantly increased.

### 3.5. Mechanism of Multifunctional Coatings

#### 3.5.1. FT-IR and UV-Vis Analysis

**Figure 7:**
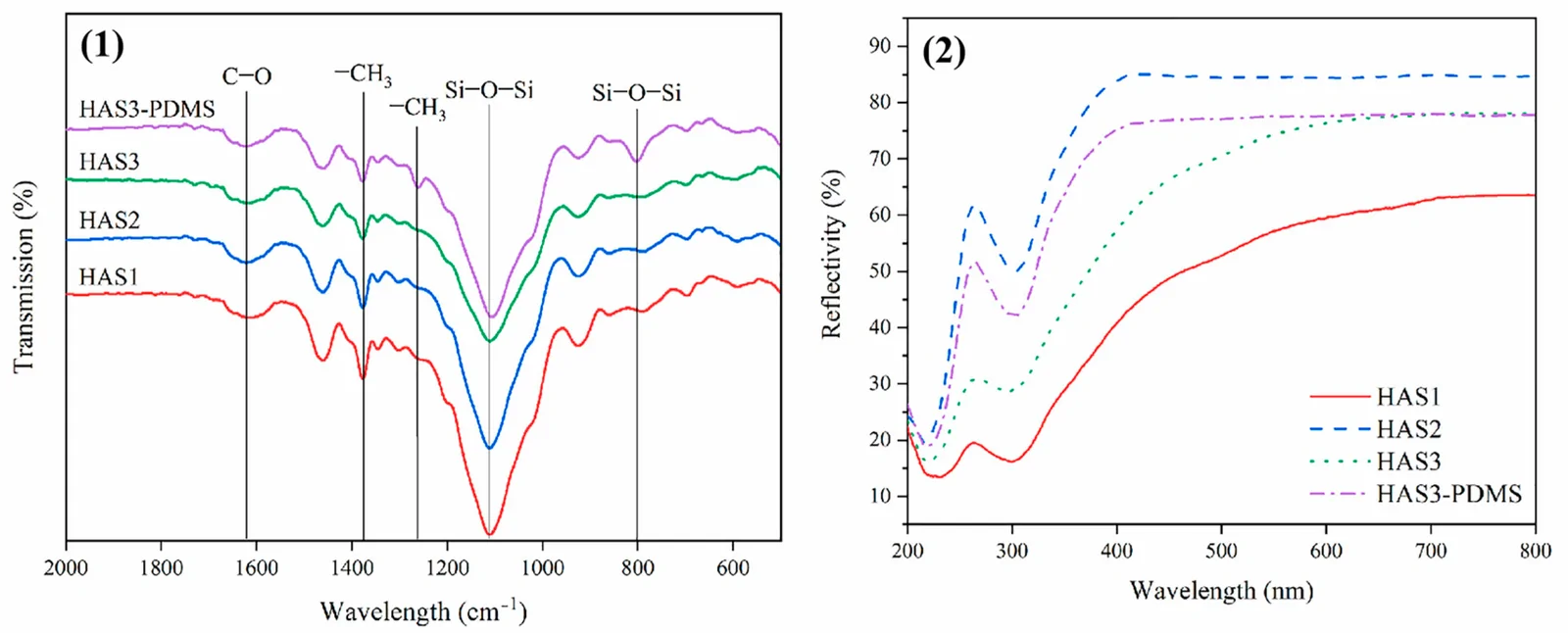
FT-IR spectra (**1**) and UV-Vis spectra (**2**) of multifunctional coatings.

FT-IR spectroscopy was conducted to investigate the structure of multifunctional coatings. The FT-IR spectra in Figure 7(1) show that the Si-O-Si (802 cm^−1^, 1130 cm^−1^), -CH_3_ (1262 cm^−1^, 1376 cm^−1^), and C-O (1632 cm^−1^) functional groups were all observed in the multifunctional coatings. The structure of the PDMS was highly similar to that of silica and exhibited similar infrared characteristic peaks with slightly different intensities [36]. Previous reports and Kirchhoff’s law have shown that [37,38,39] the phonon polarization resonance of the Si-O-Si functional group gives silica nanoparticles strong emissivity in the atmospheric transparency window. The C-O functional group has a high emissivity in the mid-infrared band, which gives the multifunctional coatings a low sunlight absorption in related bands. Moreover, upon increasing the ratio of nanoparticles and introducing the PDMS into the hybrid coatings, the intensity of the absorption band of the -CH_3_ increased. The strong stretching vibration of the -CH_3_ functional groups resulted in low sunlight absorption and helped improve the hydrophobicity of the multifunctional coatings. These findings are consistent with the temperature measurements and WCA results, which indicate that the simultaneous presence of the Si-O-Si, -CH_3,_ and C-O functional groups made the multifunctional coatings ideal materials with both heat-shielding properties and hydrophobicity.

The UV reflectance spectra of the multifunctional coatings are shown in Figure 7(2). When exposed to air and light (especially UV light), polymers such as PET are susceptible to photooxidation, which degrades their polymer chains and affects their mechanical and thermal properties. The reflectivity of the multifunctional coatings at wavelengths of 200−800 nm increased upon increasing the content of the nanoparticles, and the highest reflectivity at 800 nm was 81%. In comparison, the highest reflectivity of HAS1 was 63% at 800 nm. In addition, the introduction of PDMS significantly increased the reflectivity at wavelengths of 250−400 nm, which is the main UV-absorbing region of PET [40]. These results can be used to improve the photoaging resistance of multifunctional coatings and further explain the changes in the WCAs during the aging test. This was due to the synergistic effects of SiO_2_ and Al_2_O_3_ nanoparticles, which provided an excellent shielding effect, and the cross-linking of the modified nanoparticles and PDMS, which caused the coating’s structure to be packed closely [41].

#### 3.5.2. XPSAnalysis

**Figure 8:**
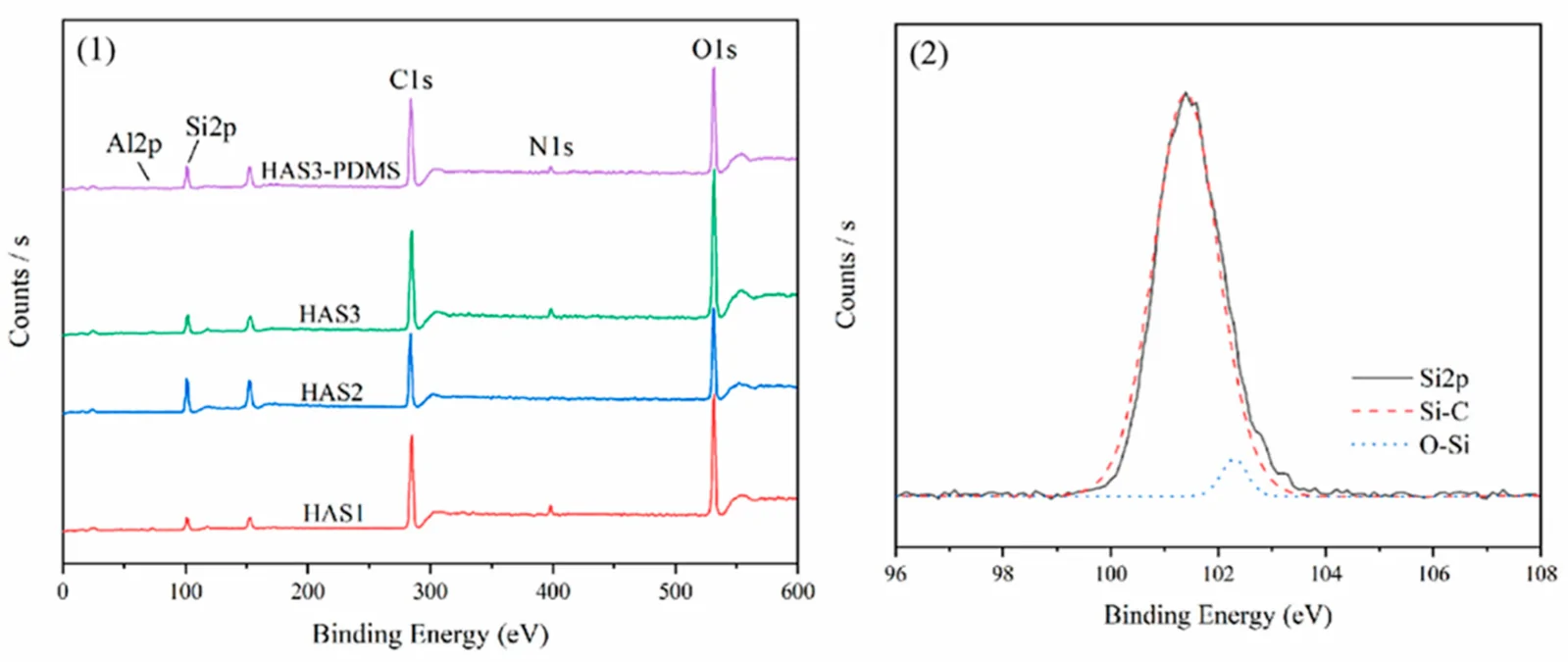
XPS full spectra of multifunctional coatings (**1**); high-resolution Si2p spectrum of HAS3-PDMS (**2**).

XPS was conducted to explore the chemical compositions of multifunctional coatings. As shown in Figure 8(1), Si, C, and O peaks were detected with accompanying N and Al peaks in all samples. The Si 2p high-resolution spectra revealed the presence of two main peaks for the Si species (Figure 8(2)) at 101.5 eV for the Si-C bonding configurations and at 102.3 eV for the O-Si bonding configurations [42]. Si-C bonds are commonly related to methyl (-CH_3_) functional groups, while Si-O bonds are characteristic of the SiO_2_ network and also related to the main chains of PDMS [43].

The atomic percentages and atomic ratios for various species in the multifunctional coatings are listed in Table 4. The amount of curing agent was sufficient for all experimental groups. Therefore, the degree of cross-linking was mainly evaluated by the atomic ratio of C/(Si + Al), where a lower C/(Si + Al) ratio generally indicates a higher degree of cross-linking [44]. Comparing the atomic ratio across the samples showed that HAS1 had the highest value of 6.37. The ratio of HAS3-PDMS was slightly lower than that of HAS3, which was consistent with the high-resolution spectra, which showed that the O-Si bonds were also related to PDMS. This indirectly suggested the self-enrichment of PDMS on the coating surface. This was also consistent with the WCA, which showed that the introduction of PDMS with a low surface energy increased the hydrophobicity of the coating surface. In addition, the filling of gaps between the nanoparticles by the PDMS also helped densify the coating. HAS2 had the lowest ratio of 2.16, indicating that pretreatment with KH560 improved the degree of cross-linking via epoxy-amine reactions. Therefore, increasing the content of nanoparticles and pretreatment with KH560 significantly improved the degree of cross-linking, which played a positive role in enhancing the hardness and heat-shielding properties of the multifunctional coatings.

## 4. Conclusions

This study developed a controllable method to fabricate multifunctional transparent and flexible yet hard coatings with weathering resistance and heat-shielding properties based on HMDS-SiO_2_, KH560-SiO_2_, KH560-Al_2_O_3,_ and PDMS. By varying the type and composite ratio of the nanoparticles, the structure and properties of the coatings could be designed to meet different application requirements without additional UV irradiation. A clear coating with a hardness of 4H, a flexibility of 10 mm, low thermal conductivity of 0.3827 W/(m·K), a temperature reduction of 7.3 °C, and a transmittance larger than 97% was obtained. Owing to the synergy of the intrinsic optical properties of nanoparticles, the multifunctional coatings exhibited a high reflectivity (81%), which should help improve their weathering resistance and heat-shielding properties. The introduction of PDMS also significantly improved the hydrophobicity (WCA = 141.37°) due to its self-enrichment on the surface of coatings.

## Figures and Tables

**Table 1:** Formulations of multifunctional coatings.

Samples	Formulation (g)				
	HMDS-SiO2	KH560-SiO2	KH560-Al2O3	D400	PDMS
HAS1	0.5	0.2	0.3	10	/
HAS2	0.6	0.4	1.0	10	/
HAS3	1.0	0.4	0.6	10	/
HAS3-PDMS	1.0	0.4	0.6	7	3

**Table 2:** Hardness, adhesion, and flexibility of multifunctional coatings.

Samples	Hardness	Adhesion		Ra (µm)	Flexibility
		Glass	PET		
HAS1	2H	1	2	2.417	10 mm
HAS2	4H	0	1	5.733	10 mm
HAS3	4H	0	1	5.207	10 mm
HAS3-PDMS	3H	1	2	4.610	10 mm

**Table 3:** Transmittance and haze of the coated glass.

Samples	Transmittance (%)	Haze (%)
HAS1	99.6	1.57
HAS2	98.8	8.50
HAS3	98.8	5.03
HAS3-PDMS	97.5	10.24

**Table 4:** Atomic percentages and atomic ratios of multifunctional coatings.

Samples	Atomic (%)					Atomic Ratio
	Al2p	Si2p	C1s	N1s	O1s	C/(Si + Al)
HAS1	2.23	6.95	58.45	4.16	28.21	6.37
HAS2	1.24	21.34	48.72	1.33	27.38	2.16
HAS3	1.99	8.78	54.70	3.88	30.65	5.08
HAS3-PDMS	1.34	12.10	56.83	2.61	27.12	4.23

## Data Availability

The raw data supporting the conclusions of this article will be made available by the authors on request.
